# Supplementary material for: The Biological Properties of the SARS-CoV-2 Cameroon Variant Spike: An Intermediate between the Alpha and Delta Variants
Source: Pathogens. 2022 Jul 20;11(7):814. doi: 10.3390/pathogens11070814 (PMC9315702; doi:10.3390/pathogens11070814)
Supplement: Supplementary file 1 [file pathogens-11-00814-s001.zip › pathogens-1772637-supplementary.pdf]

Supplementary Table S1. Mutations of the Spike NTD of variants with respect to the reference Wuhan Spike, mutation ΔΔG and domain net charge at pH=7.00 (last table line).

Alpha	Delta	ΔΔG (kcal/mol)	Omicron	ΔΔG (kcal/mol)	B.1.640.1	ΔΔG (kcal/mol)	IHU B.1.640.2	ΔΔG (kcal/mol)
Δ69-70	T19R	-0.53	A67V	-1.62	P9L		P9L	
Δ144	G142D	5.66	Δ69-70	–	E96Q	1.32	E96Q	1.32
	E156G	3.67	T95I	-1.65	Δ136-144	4.31[a]	Δ136-144	4.31[a]
	Δ157-158		G142D	5.91	R190S	3.97	R190S	3.97
			Δ143-145	–	I210T	2.87	D215H	2.03
			Δ211	–				
			L212I	0.27				
			+214EPE	–				
1.2	1.6		-2.0		2.6		3.7	

a) This value refers to the substitution C136A that simulates the removal of the disulfide bridge upon deletion of the region 136-144.

Supplementary Table S2. B.1.640.1 mutations occurring in the Spike protein with respect to the reference Wuhan Spike protein. Structural context and interaction changes (added or removed) compared to those found in the reference 7SY1 Spike.

**Mutations[a]**	**Structural context**	**Changed interactions**
E96Q	NTD; loop connecting two β-strands	Removes the salt bridge with R190
Δ136-144	NTD: strand of a β-hairpin	Removes a β-strand and the disulfide bridge C15-C136. Possibly interacting with the AXL receptor
R190S	NTD; within the β-strand encompassed by positions 188-197	Removes the salt bridge with E96
I210T	NTD: loop connecting two β-strands	H-bond between side chain -OH and peptide CO of F186
R346S	RBD: loop connecting two α-helices	
N394S	RBD: loop at the interface with the NTD of the other chain	
**Y449N**	RBD: loop connecting a short α-helix and a β-strand.	Removes H-bond with ACE2 D38
**F490R**	RBD: within a loop	Possible weak salt bridge with ACE2 E35
**N501Y**	RBD	Interaction with ACE2 Y41 and K353
D614G	S1	
P681H	S1: Exposed loop not visible in the reference structure.	
T859N	S2: within a β-strand at the interface with the other subunit.	Forms a H-bond with N317 of the other subunit
D936H	S2 HR1: exposed side of an α-helix	

a) Residues at the interface with ACE2 are boldfaced

Supplementary Table S3. Variant mutations occurring in the Spike RBD with respect to the reference Wuhan Spike protein, ΔΔG calculated with FoldX, and domain net charge at pH=7.00 (table last line). Text color marks shared mutations. Grey background denotes mutations at the RBD interface to ACE2. The mutation E484K is highlighted with blue, bold text.

**Alpha**	**ΔΔG (kcal/mol)**	**Delta**	**ΔΔG (kcal/mol)**	**Omicron**	**ΔΔG (kcal/mol)**	**B.1.640.1**	**ΔΔG (kcal/mol)**	**IHU B.1.640.2**	**ΔΔG (kcal/mol)**
**N501Y**	1.2	L452R	0.2	G339D	-1.0	R346S	1.1	R346S	1.1
		**T478K**	-0.2	S371L	-0.3	N394S	0.4	N394S	0.4
				S373P	4.2	Y449N	0.2	Y449N	0.2
				S375F	-0.3	F490R	1.2	**E484K**	0.5
				K417N	-0.2	**N501Y**	2.0	**F490S**	2.1
				N440K	-0.4			**N501Y**	2.0
				G446S	3.0				
				S477N	0.1				
				**T478K**	-0.2				
				E484A	1.0				
				Q493R	0.2				
				G496S	-0.9				
				Q498R	0.3				
				**N501Y**	1.2				
				Y505H	0.7				
2.6		4.1		5.2		2.2		3.2	

Supplementary Table S4. Comparison of the alanine scanning of the RBD-ACE2 complexes. The loss of interaction energy (ΔΔG) upon substitution of each interface residue with alanine is reported. Only mutated residues are displayed for B.1.640.1 and 2.

**Alpha (B.1.1.7)**	**ΔΔG (kcal/mol)**	**B.1.640.1**	**ΔΔG (kcal/mol)**	**IHU B.1.640.2**	**ΔΔG (kcal/mol)**
R403	0.11		0.11		0.11
K417	0.19		0.19		0.19
V445	0.03		0.03		0.03
Y449	0.79	N	0.00	N	0.16
Y453	0.86		0.84		0.84
L455	0.42		0.42		0.42
F456	0.55		0.57		0.57
Y473	0.22		0.25		0.25
S477	0.05		0.05		0.05
E484	0.12		0.15	K	0.10
F486	0.86		0.85		0.85
N487	1.22		1.25		1.25
Y489	2.30		2.16		2.16
F490	0.10	R	0.11	S	0.05
Q493	1.05		1.06		1.01
S494	0.09		0.13		0.18
Y495	0.09		0.09		0.09
Q498	0.57		0.57		0.57
T500	0.40		0.32		0.32
Y501	3.35		3.18		3.18
V503	0.03		0.03		0.03
Y505	3.81		3.79		3.79
